# Stable isotopes in bivalves as indicators of nutrient source in coastal waters in the Bocas del Toro Archipelago, Panama

**DOI:** 10.7717/peerj.2278

**Published:** 2016-08-02

**Authors:** Lauren E. Graniero, Ethan L. Grossman, Aaron O’Dea

**Affiliations:** 1 Department of Geology and Geophysics, Texas A&M University, College Station, TX, USA; 2 Smithsonian Tropical Research Institute, Balboa, Republic of Panama

**Keywords:** Anthropocene, Caribbean, Eutrophication, Molluscs, Nitrogen isotopes, Carbon isotopes

## Abstract

To examine N-isotope ratios (^15^N/^14^N) in tissues and shell organic matrix of bivalves as a proxy for natural and anthropogenic nutrient fluxes in coastal environments, *Pinctada imbricata*,* Isognomon alatus*, and *Brachidontes exustus*bivalves were live-collected and analyzed from eight sites in Bocas del Toro, Panama. Sites represent a variety of coastal environments, including more urbanized, uninhabited, riverine, and oceanic sites. Growth under differing environmental conditions is confirmed by *δ*^18^O values, with open ocean Escudo de Veraguas shells yielding the highest average *δ*^18^O (−1.0‰) value and freshwater endmember Rio Guarumo the lowest (−1.7‰). At all sites there is no single dominant source of organic matter contributing to bivalve *δ*^15^N and *δ*^13^C values. Bivalve *δ*^15^N and *δ*^13^C values likely represent a mixture of mangrove and seagrass N and C, although terrestrial sources cannot be ruled out. Despite hydrographic differences between end-members, we see minimal *δ*^15^N and *δ*^13^C difference between bivalves from the river-influenced Rio Guarumo site and those from the oceanic Escudo de Veraguas site, with no evidence for N from open-ocean phytoplankton in the latter. Populated sites yield relative ^15^N enrichments suggestive of anthropogenic nutrient input, but low *δ*^15^N values overall make this interpretation equivocal. Lastly, *δ*^15^N values of tissue and shell organic matrix correlate significantly for pterioideans *P. imbricata* and *I. alatus*. Thus for these species, N isotope studies of historical and fossil shells should provide records of ecology of past environments.

## Introduction

Deforestation of rainforest and mangrove forest, pollution from industrial farming, and wastewater influx from a growing human population are contributing to the eutrophication of coastal ecosystems in the Bocas del Toro Archipelago of Panama (Cramer, 2013; Aronson et al., 2014; Schlöder, O’Dea & Guzmán, 2013). This anthropogenic influence creates changes in nutrient conditions and habitat that result in significant detrimental impacts on coastal marine biodiversity, fisheries, and tourism (e.g., Jackson, 2001; Jackson et al., 2001; Lin & Dushoff, 2004; Seemann et al., 2014). In Bocas del Toro especially, there is a correlation between high runoff and high chlorophyll-*a* concentrations (D’Croz, Rosario & Gondóla, 2005). Identifying both natural and anthropogenic nutrient sources to the coastal waters may aid in efforts to reduce eutrophication in the area. One way to characterize spatial and temporal variations in carbon and nitrogen sources to this and other coastal marine ecosystems is through the stable isotopes that are assimilated by, and accumulate in, coastal marine organisms (e.g., Torres-Rojas et al., 2014; Fertig, Carruthers & Dennison, 2014).

The carbon and nitrogen isotopic compositions in the tissues of marine organisms are a function of the isotopic compositions of the food source, as well as fractionation associated with biochemical processes (Minagawa & Wada, 1984; Peterson & Fry, 1987). Carbon and nitrogen isotope compositions are reported in *δ* notation. This is expressed as

(1) \begin{eqnarray*}{\delta }^{15}\mathrm{N}(\permil )= \left( \frac{{R}_{x}-{R}_{\mathrm{std}}}{{R}_{\mathrm{std}}} \right) \times 1000\end{eqnarray*}

where *R* is the isotopic ratio (e.g., ^13^C/^12^C, ^15^N/^14^N, or ^18^O/^16^O) of the sample (*R*_*x*_) and the standard (*R*_std_). The *δ*^13^C and *δ*^15^N values of tissue depend on various parameters such as carbon or nitrogen concentration, growth rate, trophic level, tissue composition (e.g., protein versus lipids), and food source. Shell *δ*^13^C values primarily reflect *δ*^13^C_DIC_, although a small percentage of metabolic carbon (C_*M*_) is also incorporated into the shell (Gillikin et al., 2007; McConnaughey & Gillikin, 2008). In contrast, *δ*^15^N increases by ∼3.4‰ at each trophic level because of preferential excretion of ^15^N-depleted nitrogen in urine (Minagawa & Wada, 1984; Peterson & Fry, 1987). In filter feeders like most bivalves, food source can vary temporally. This can impart a time-dependent isotopic signal that may be integrated differently depending on the turnover rate of the tissue analyzed (Carmichael et al., 2008; Torres-Rojas et al., 2014).

Nitrogen isotopes also can be effective tracers for nitrogen sources and cycling in marine and terrestrial environments. Terrestrial nitrogen is derived from many different sources and undergoes many chemical processes, nevertheless it is often distinguishable from marine nitrogen (Fertig, Carruthers & Dennison, 2014). Nitrate from the decomposition of human and animal waste has unusually high *δ*^15^N values (*δ*^15^N = 10 to 22‰; Kreitler, 1975; Heaton, 1986) due to volatilization of ammonia and microbial processes such as denitrification (e.g., Kreitler, 1975; McClelland, Valiela & Michener, 1997; Montoya, 2007; Fertig et al., 2010; Torres-Rojas et al., 2014; Fertig, Carruthers & Dennison, 2014). A study of contrasting tropical coastal environments showed that the *δ*^15^N of groundwater nitrate from an urbanized site (Jobos Bay, Puerto Rico) had groundwater values up to 12‰, indicative of wastewater nitrogen, whereas an undeveloped site (Playa Limon, Panama) had more natural values around −3‰ (Bowen & Valiela, 2008).

The large nitrogen isotope fractionations associated with microbially-facilitated reactions enrich and complicate the application of nitrogen isotopes in marine systems. For instance, denitrification preferentially consumes ^15^N-depleted nitrate, resulting in fractionations of 25–40‰, whereas nitrification of ammonia can impart a fractionation of about −35‰ (e.g., Cline & Kaplan, 1975; Mariotti et al., 1981). Of course, in substrate-limited environments, complete conversion of ammonia or nitrate erases any fractionation effects due to nitrification or denitrification.

Carbon isotopes are used in combination with nitrogen isotopes to trace sources of organic carbon input to coastal ecosystems. In Brazil, marine invertebrates and fish had *δ*^13^C values (−17.4 to −12.7‰) much higher than those of terrestrial organic matter entering the ecosystem (−29.4 ± 0.3‰), indicating organic carbon sourced from local marine particulate organic matter (POM; −18.6 ± 0.5‰) and macroalgae (−15.5 ± 1.8‰; Corbisier et al., 2006). An isotopic study of Caribbean estuarine ecosystems found phytoplankton *δ*^13^C values from Jamaica’s Hunts Bay to be slightly more negative than average marine POM at −23.1‰ (Andrews, Greenway & Dennis, 1998), reflecting minor assimilation of terrestrially-derived carbon.

In this study we evaluate nitrogen, carbon, and oxygen isotope ratios in three bivalve species to better understand habitat and nutrient dynamics in tropical coastal environments. Four specific objectives are: (1) ascertain whether N and C isotopes can be used in combination to identify the primary carbon and nitrogen sources consumed in tropical coastal environments; (2) determine if N and C isotopes can distinguish between anthropogenically-influenced and uninhabited sites; (3) validate the use of shell *δ*^15^N values as a proxy for tissue *δ*^15^N values in pterioideans; and (4) verify that shell *δ*^18^O values correlate with salinity and thus inferred environments. Our results demonstrate that shell *δ*^15^N values are a reliable proxy for bivalve tissue *δ*^15^N values in pterioideans *P. imbricata* and *I. alatus*. They also determine that bivalve *δ*^15^N and *δ*^13^C values primarily reflect local mangrove and seagrass sources at all sites, although terrestrial influences cannot be ruled out.

## Hydrologic Setting

**Figure 1:**
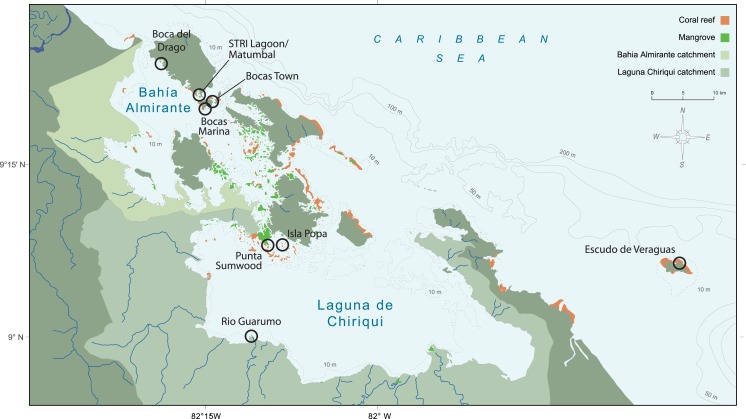
Map of the Bocas del Toro Archipelago, Panama. Almirante Bay and Chiriquí Lagoon watersheds are represented by green shading. Reef extent is represented by orange shading and mangrove extent is represented by light green shading. Sample sites are labeled with black circles.

The Bocas del Toro Archipelago is comprised of two oceanographic features, Almirante Bay (446 km^2^) and the significantly larger Chiriquí Lagoon (941 km^2^; D’Croz, Rosario & Gondóla, 2005; Fig. 1). These semi-enclosed areas are bordered by mangroves and have restricted exchange with Caribbean waters (Quiroz et al., 2011). Seasonal variations in freshwater inputs to Almirante Bay and Chiriquí Lagoon is dominated by runoff and rainfall regimes. Almirante Bay is exposed to the Caribbean Sea near Boca del Drago. Almirante Bay also has small outlets near Isla Colón and Isla Popa. Chiriquí Lagoon is exposed to the Caribbean Sea near Cayo Agua, however the lagoon receives significant freshwater from the Cricamola River, Rio Guarumo, and numerous others (Quiroz et al., 2011). Chiriquí Lagoon’s drainage basin is also significantly larger than that of Almirante Bay (Fig. 1; Quiroz et al., 2011). Thus, despite being better connected to the open ocean, Chiriquí Lagoon is more turbid and nutrient-rich (N, P, Si) than Almirante Bay (D’Croz, Rosario & Gondóla, 2005; Kaufmann & Thompson, 2005). Because of very low nutrient concentrations in local marine waters, river discharge is the primary control on nutrient content in both Almirante Bay and Chiriquí Lagoon (D’Croz, Rosario & Gondóla, 2005).

## Sample Collections

Five live specimens of bivalves *Pinctada imbricata* (Röding, 1798), *Isognomon alatus* (Gmelin, 1791), and *Brachidontes exustus* (Linnaeus, 1758) were hand-collected in July 2013 from mangrove roots or docks at eight locations in the Bocas del Toro Archipelago, Panama (Fig. 1). Collection permits were approved by the Autoridad de los Recursos Acuáticos de Panamá (permiso con fines científicos N°55). Previous studies indicate five individuals are the optimum sample size for isotopic studies of bivalves as bioindicators (Fertig et al., 2010). The study sites target locations with varying degrees of development and a range of salinities. Escudo de Veraguas, a mature forested island that is virtually uninhabited, serves as an open-ocean end-member, whereas the Rio Guarumo site is most affected by river runoff (Fig. 1). Additional study sites span a range of land use types. The most populated sites are Bocas Town and Bocas (Town) Marina, which are characterized by developed land use with a notable percentage of impervious surfaces (e.g., roads, buildings, etc.) interspersed with mangroves. These urban sites are known to be affected by high sedimentation rates and urban runoff (Hilbun, 2009). Intermediately impacted sites Boca del Drago and STRI Lagoon (Matumbal) are semi-enclosed lagoons draining logged forests and stubble boarded by mangroves. Minimally impacted sites Punta Sumwood and Isla Popa sites are characterized by logged forest and stubble, mangroves, and subsistence agriculture.

## Sample Preparation and Analysis

Water temperature, conductivity, and dissolved oxygen (DO) were measured at the time of collection using a YSI Pro 2030 multiparametric sonde from sample sites at approximately 0.5 m water depth (Table 1).

**Table 1:** Physical characteristics of the eight sample sites in Bocas del Toro, Panama at the time of sampling. Dashes occur where data were not collected.

	Escudo de veraguas	Punta sumwood	Isla popa	Rio guarumo	Boca del drago	STRI facility	Bocas town	Bocas town marina	Avg.	Standard deviation
DO (mg/L)	5.3	5.5	3.9	4.4	5.2	6.7	5.1	4.4	5.0	0.8
DO%	83	80	57	68	78	104	78	67	77	13
T (°C)	28.5	28.1	28.7	29.3	28.2	30.0	28.8	28.7	28.8	0.6
SPC (mS/cm)	51.8	47.9	48.5	–	48.4	48.1	49.2	49.7	49.1	1.3
Sal (ppt)	34.0	31.1	31.5	31.6	31.5	31.3	32.0	32.4	31.9	0.9
Mangroves (Y/N)	Y	Y	Y	Y	Y	Y	N	Y	–	–
Seagrass (Y/N)	Y	N	Y	–	Y	–	N	–	–	–
Algae (Y/N)	Y	N	Y	–	Y	Y	Y	Y	–	–
Species available (I, P, Br)	P	I, P	I, P, Br	P, Br	I, P, Br	I, P, Br	I, P	I, P	–	–

The bivalves were dissected and tissues were separated by gill, muscle, mantle, and stomach, and subsequently dried overnight at 60–65°C. Samples were then sealed in airtight microcentrifuge tubes and crushed and homogenized using a mortar and pestle. Approximately 1 mg of the powdered sample was analyzed for *δ*^13^C and *δ*^15^N using a Carlo Erba NA1500 elemental analyzer (EA) coupled to a Thermo Finnigan Delta^plus^XP isotope ratio mass spectrometer (IRMS) at the Stable Isotope Geosciences Facility at Texas A&M University (http://geosciences.tamu.edu/facilities/stable-isotope-geosciences-facility/index.php). At least every fifth sample was run in duplicate. Carbon and nitrogen isotope values were calibrated with USGS40 (−26.39‰ and −4.52‰ respectively) and USGS41 (37.63‰ and 47.57‰ respectively) L-glutamic acid standards, and reported versus VPDB and air respectively. Analytical precision was 0.08‰ for *δ*^15^N and 0.13‰ for *δ*^13^C based on replicates of standards. Conventionally, studies measure the *δ*^15^N of muscle and/or mantle tissue, therefore these values are averaged for *δ*^15^N_tissue_ versus *δ*^15^N_shell_ comparisons in this paper.

Bivalve shells were cleaned by lightly sanding and scrubbing with dilute soap and water to remove surface contaminants. Using the lowest speed (5000/min) on a Dremel 3000 tool carbonate powder was milled on the shell exterior, parallel to the direction of growth, to obtain an average *δ*^15^N value for each shell. For small shells, the entire outer layer of the shell was removed and homogenized with a mortar and pestle. Care was taken to avoid the inner aragonitic (nacreous) shell layer. Roughly 40–100 µg of powdered shell was analyzed for *δ*^13^C and *δ*^18^O using a Thermo Scientific MAT 253 IRMS coupled to a Kiel IV automated carbonate reaction system. For *δ*^15^N, 5 mg of powdered shell was analyzed using the same EA-IRMS system mentioned above. The only difference is that sample gas was passed through a NaOH/silica trap to remove CO_2_ due to the relatively small amount of nitrogen in the shell compared with carbon. Carbon and oxygen isotope analyses were calibrated using the NBS-19 standard (*δ*^13^C = 1.95‰; *δ*^18^O = −2.20‰) and reported versus VPDB. Nitrogen isotope calibration is the same as above. At least every fifth sample was run in duplicate. Analytical precision was 0.11‰ for *δ*^15^N, 0.06‰ for *δ*^13^C, and 0.08‰ for *δ*^18^O based on replicates of standards.

**Figure 2:**
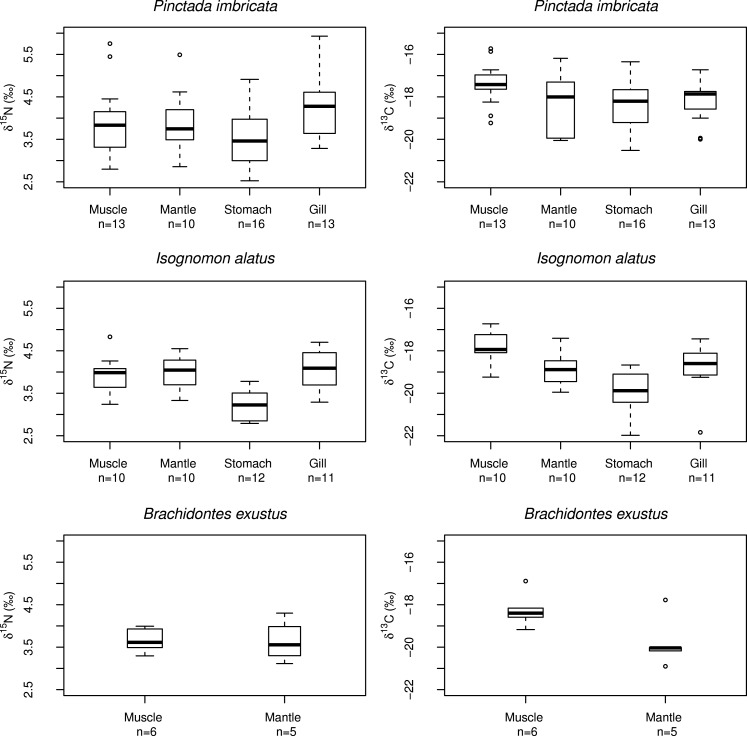
Boxplots of muscle, mantle, stomach, and gill tissue *δ*^15^N and *δ*^13^C values including all locations for *P. imbricata*, *I. alatus*, and *B. exustus*.

## Results

### Tissue *δ*^13^C and *δ*^15^N values

Analysis of variance indicated that at 95% confidence, there was no significant difference between mean *δ*^15^N values (*F* = 2.69, *df* = 3, *p* = 0.06) or mean *δ*^13^C values (*F* = 2.34, *df* = 3, *p* = 0.09) between tissue types for *P. imbricata*, however there was a significant difference in mean *δ*^15^N values (*F* = 10.44, *df* = 3, *p*<0.001) and mean *δ*^13^C values (*F* = 8.55, *df* = 3, *p* < 0.001) between tissue types for *I. alatus* (Fig. 2). There were significant taxonomic differences between *P. imbricata* and *I. alatus* in average *δ*^13^C_tissue_ values (*Welch’s *t*-test*, *t* = 3.33, *df* = 88.30, *p* < 0.01; Fig. 2). However, there was no significant difference between *P. imbricata* and *I. alatus* average *δ*^15^N_tissue_ values (*Welch’s *t*-test*, *t* = 0.81, *df* = 89.89, *p* < 0.42). For *B. exustus*, tissue *δ*^15^N values were not significantly different between mantle and muscle tissues (*Welch’s *t*-test, t* = 0.02, *df* = 5.95, *p* = 0.98), but mean mantle and muscle *δ*^13^C values were significantly different (*Welch’s *t*-test*, *t* = 2.48, *df* = 6.64, *p* < 0.05).

**Figure 3:**
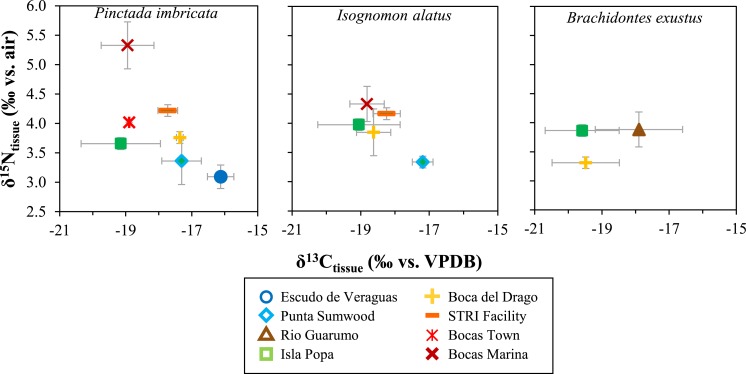
Location averages for muscle and mantle tissue *δ*^15^N versus *δ*^13^C for each respective location. Error bars shown are ±2 SE of replicate samples for each site.

Considering all species and tissue types, Bocas Marina tissue *δ*^15^N values range from 2.5 to 6‰, Boca del Drago values range from 3 to 4.5‰, and STRI Facility values range from 3.5 to 5.3‰ (Appendix S1). In the case of Bocas Marina, this range is roughly equivalent to an increase in trophic level (∼3‰). Narrowing the focus to muscle and mantle tissues decreased variability substantially.

**Figure 4:**
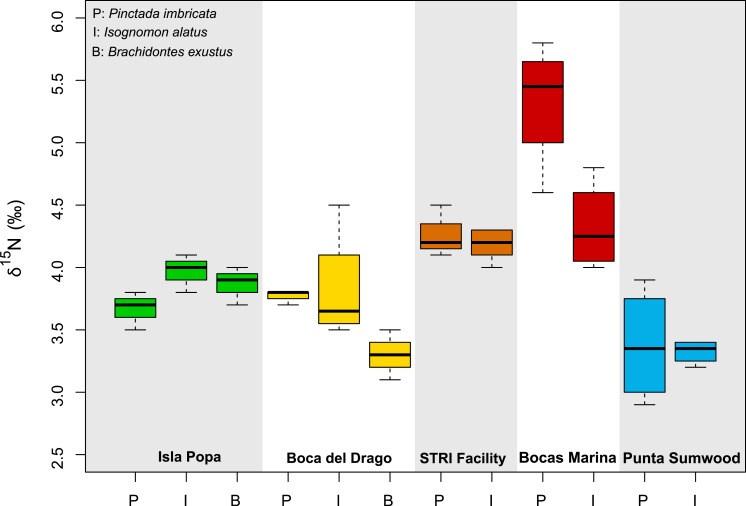
Boxplots displaying variability between different species at study sites where two or more species were present.

Between study sites there were no consistent taxonomic trends between *P. imbricata* and *I. alatus* in mean *δ*^13^C_tissue_ or *δ*^15^N_tissue_ values (Fig. 3). For *δ*^15^N in muscle and mantle tissues, some sites show notable differences between *P. imbricata* and *I. alatus* species (Bocas Marina, Isla Popa,), but not others (Punta Sumwood, STRI Facility, Boca del Drago) (Fig. 4). Open-ocean end-member Escudo de Veraguas is unpopulate except for low numbers of seasonal fisherman and has a distinctly low average *δ*^15^N_tissue_ value (∼3‰), in contrast with more populated sites (≥4‰).

Bivalve tissue *δ*^13^C values from all sites range from about −21 to −16‰ (Fig. 3). Escudo de Veraguas specimens had a high *δ*^13^C value (∼ − 16‰) compared with human occupied sites (<−17.5‰) (Fig. 3). The *δ*^15^N and *δ*^13^C differences between the open-ocean Escudo de Veraguas site and the human occupied sites drive a negative correlation between *δ*^15^N_tissue_ and *δ*^13^C_tissue_ values in *P. imbricata* (*R*^2^ = 0.40) and *I. alatus*(*R*^2^ = 0.60), the taxa best represented in the sample set (Fig. 3).

### Shell *δ*^15^N values and tissue-shell comparisons

**Figure 5:**
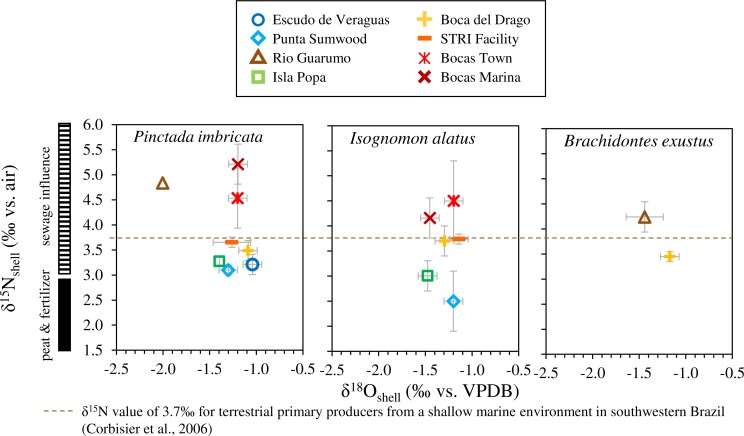
Location averages for muscle and mantle tissue *δ*^15^N versus *δ*^13^C for each respective location. Error bars shown are ±2 SE of replicate samples for each site.

Bivalve shells analyzed from urban sites (Bocas Town and Bocas Town Marina) had the highest *δ*^15^N_shell_ values on average (all species), 4.5‰ and 4.7‰, respectively (Fig. 5). The river-influenced Rio Guarumo site had the next highest average *δ*^15^N values (4.5‰). STRI Facility and Boca del Drago had intermediate *δ*^15^N values of 3.7‰ and 3.6‰, respectively. Isla Popa has a low average *δ*^15^N value of 3.1‰, similar to open-ocean end-member Escudo de Veraguas (3.2‰). The site with the lowest *δ*^15^N value was Punta Sumwood (2.8‰). There were no significant differences in shell *δ*^15^N values between *P. imbricata* and *I. alatus* at all sites except Bocas Marina (*Welch’s *t*-test, t* = − 3.78, *df* = 8.00, *p* < 0.05).

**Figure 6:**
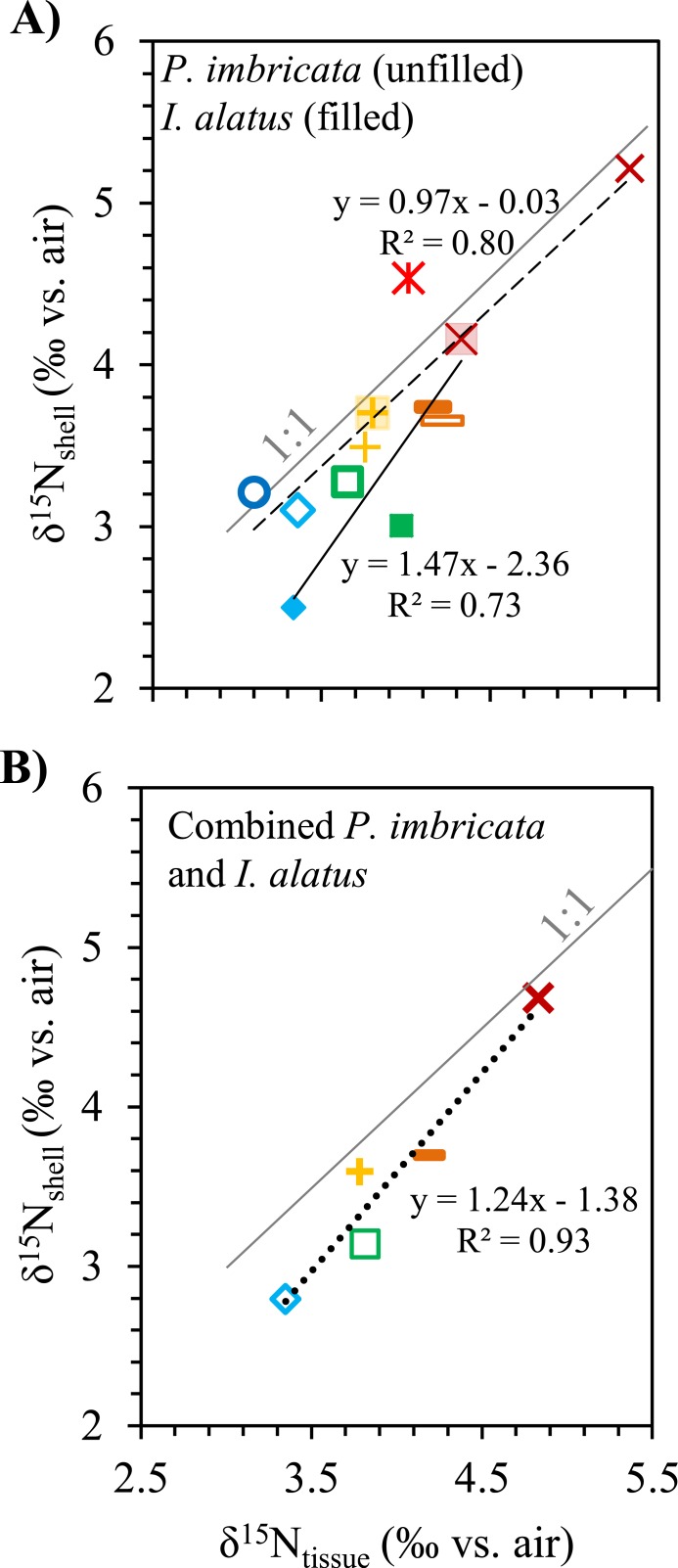
*δ*^15^N comparison for muscle and mantle tissues and corresponding species average shell value for (A) *P. imbricata*, * I. alatus*, and (B) combined (compared to average shell values for both species). * Brachidontes exustus* were not available in enough abundance for statistical analysis. Each point represents the average value of specimens at one location.

On average, *P. imbricata* and *I. alatus* shell contained 0.01% N, while *B. exustus* contained 0.06% N. Average *δ*^15^N values in shells co-vary with, and are statistically identical to, those in tissues for pterioideans *P. imbricata* and *I. alatus* (Fig. 6). Muscle and mantle tissue *δ*^15^N values were not significantly different from *B. exustus* shell *δ*^15^N values. Correlations between *δ*^15^N_tissue_ and *δ*^15^N_shell_ values were significant for *P. imbricata*, *I. alatus*, and combined *P. imbricata* and *I. alatus* (Fig. 6).

### Shell *δ*^18^O and *δ*^13^C values

**Figure 7:**
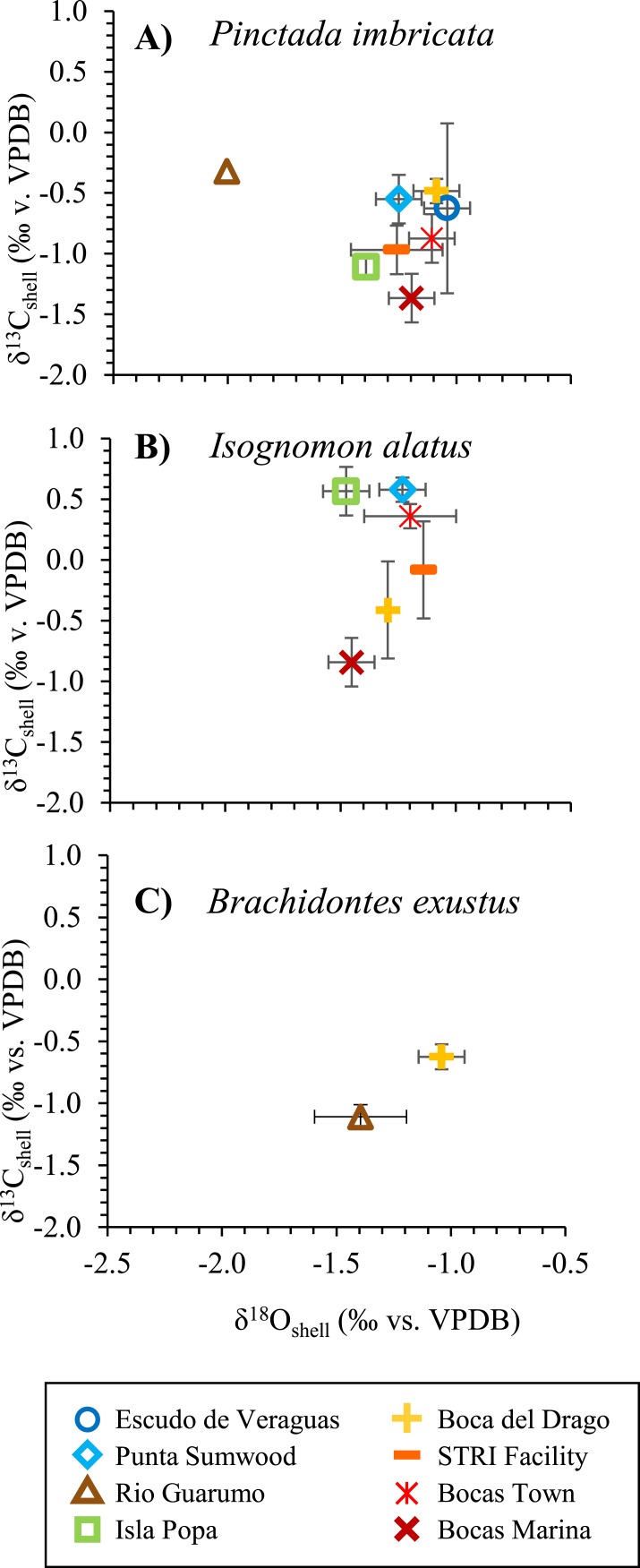
Average shell *δ*^13^C versus *δ*^18^O values shell carbonate values for each location. Average shell *δ*^13^C versus *δ*^18^O values shell carbonate values for each location including (A) *Pinctada imbricata*, (B) *Isognomon alatus*, and (C) *Brachidontes exustus* (*n* = 73). Location symbols are the same for remaining figures unless stated otherwise. Ti, tissue; Sh, shell. Error bars shown are ±2 SE of replicate samples for each site.

The average shell *δ*^18^O values for each species from each site range from −1.0 to −2.0‰ (Fig. 7). The open-ocean site at Escudo de Veraguas has the highest average *δ*^18^O value (−1.0‰), whereas the Rio Guarumo site yields the lowest (−1.5‰; Figs. 2A and 2C). The Isla Popa site (−1.4‰ *P. imbricata*; −1.5‰ *I. alatus*), enclosed within Chiriquí Lagoon, exhibits intermediate *δ*^18^O values. Bocas Marina shells differ in *δ*^18^O according to taxon and show intermediate values, with *I. alatus* values averaging −1.5‰ and *P. imbricata* values averaging −1.2‰. Sites Bocas Town, Boca del Drago, STRI Lagoon (Matumbal), and Punta Sumwood are all intermediate and similar in value (average −1.1 to −1.3‰). *I. alatus* and *P. imbricata* species from the same site yielded *δ*^18^O values that were not statistically different except at Bocas Marina (Fig. 7). Escudo de Veraguas and Rio Guarumo did not have any *I. alatus* specimens present.

Overall, shell *δ*^13^C values from the Bocas del Toro region range from −1.7 to 1.8‰ and do not covary with *δ*^18^O. Shell *δ*^13^C values vary by up to ∼2‰ when comparing *P. imbricata* and *I. alatus* specimens from the same site (Fig. 7). *δ*^13^C values of *I. alatus* shells are significantly different (*Welch’s *t*-test*, *p* < 0.05) than those of *P. imbricata* shells at STRI Facility, Isla Popa, and Punta Sumwood, whereas no significant differences occur at Bocas Town, Bocas Town Marina, and Boca del Drago.

## Discussion

Previous studies provide *δ*^15^N and *δ*^13^C values for potential nitrogen and carbon sources in the Bocas del Toro Archipelago. In Almirante Bay, coastal sources have *δ*^15^N and *δ*^13^C values of respectively 2.5‰ and −12.1‰ for terrestrial grass, 1.5‰ and −8.5‰ for seagrass *Thalassia testudinum*, and 1.7‰ and −26.5‰ for mangrove (*Rhizophora*) peat (Hilbun, 2009). Carbon isotope data for forest floor litter and grassland carbon, derived from Barro Colorado Island in Lago Gatun in central Panama, average −30.1 ± 0.5‰ and −16.2 ± 4.2‰ respectively (Schwendenmann & Pendall, 2006). These values help to interpret the organic matter sources indicated by bivalve *δ*^15^N and *δ*^13^C values.

Nitrogen and carbon isotope analyses of bivalve shells and tissue in the Bocas del Toro Archipelago record spatial variations in dietary sources of nitrogen and carbon. Variations in *δ*^15^N values cannot simply be explained by the degree of terrestrial or marine influence. Bivalve *δ*^15^N values for Escudo de Veraguas, the marine end-member, do not show evidence for N from open ocean POM (4–7‰; e.g., Cifuentes et al., 1996; Montes et al., 2013), especially considering trophic enrichment (3.4‰; Minagawa & Wada, 1984), but instead appear to indicate a mixture of coastal marine sources (e.g., *T. testudinum* and *R. mangle*). The Rio Guarumo site, on the other hand, should be most influenced by river processes and terrestrial N sources, being located within 50 m of the river mouth. Riverine sources of organic matter typically include vegetation from the catchment area (Bouillon & Connolly, 2009). Therefore, one would expect greater contribution of N from terrestrial primary producers (3.7‰; Corbisier et al., 2006), including terrestrial grass (2.5‰; Hilbun, 2009), forest leaf fragments (1.5 to 3‰; Hietz et al., 2011), and banana leaf fragments (5.7‰; Hilbun, 2009). Despite hydrographic differences between end-members Rio Guarumo and Escudo de Veraguas, average *δ*^15^N values differ by only 1.3‰, suggesting a similar mixture of N sources, probably reflecting the dominance of mangrove and seagrass in both. Similarly, *δ*^15^N measurements of *Thalassia* shoots show small and insignificant differences between specimens from the ocean-facing outer lagoon (3.6 ± 0.2‰, ±1 SE), Almirante Bay (3.2 ± 0.3‰), and Chiriquí Lagoon (2.5 ± 0.4‰; Carruthers et al., 2005). These *δ*^15^N values are greater than those found by Hilbun (2009), suggesting spatial and/or temporal heterogeneity in *Thalassia δ*^15^N values across the region. Samples from urbanized sites in Almirante Bay (Bocas Town, STRI Facility, Bocas Marina) have the highest values (3.7 to 5.2‰). These sites are susceptible to human influence, but are not heavily urbanized. The sites may be subject to anthropogenic nutrient influx in the form of sewage or septic waste, which could contribute N with very high *δ*^15^N (10 to 22‰; Kreitler, 1975; Heaton, 1986). The 2.0‰ difference in *δ*^15^N between *Pinctada* values from Bocas Marina and Escudo de Veraguas samples could be explained by ∼20% contribution of anthropogenic N with a *δ*^15^N of 10‰(e.g., McClelland, Valiela & Michener, 1997).

**Figure 8:**
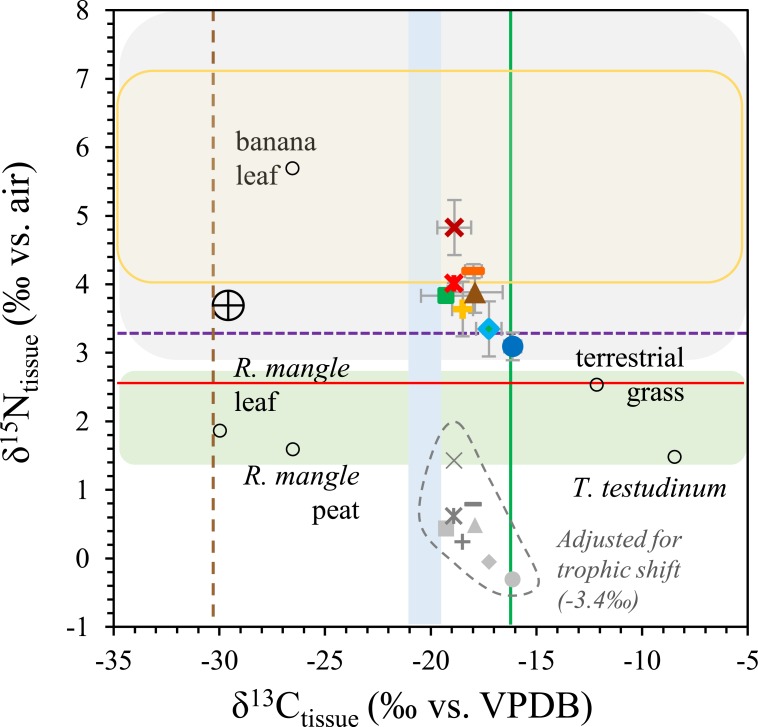
Location averages for *δ*^15^N versus *δ*^13^C including all species, compared with values for potential N and C sources. Error bars shown are ±2 SE. Symbols are defined in Fig. 2. Banana leaf, *R. mangle*, terrestrial grass and *T. testudinum δ*^15^N and *δ*^13^C values are from Almirante Bay, Panama (Hilbun, 2009). The gray-shaded rounded rectangle indicates potential *δ*^15^N values of sewage (up to 20‰; Lapointe, Barile & Matzie, 2004; McClelland, Valiela & Michener, 1997). The green-shaded rounded rectangle estimates the forest leaf range in *δ*^15^N values in legumes and non-legumes from Barro Colorado Island, Panama in 2007 (1.5 to 3.0‰; Hietz et al., 2011). The yellow rounded rectangle represents *δ*^15^N values of open ocean POM (4–7‰; Cifuentes et al., 1996; Montes et al., 2013). The blue-shaded rectangle represents coastal POM values from the Bocas del Toro Archipelago (−21.2 to −19.6‰; Seemann et al., 2014). The vertical solid green line denotes grassland *δ*^13^C values and the vertical dashed brown line denotes forest floor litter *δ*^13^C values from Barro Colorado Island, Panama (Schwendenmann & Pendall, 2006). The represents the *δ*^15^N value of terrestrial organic matter entering a coastal ecosystem in Brazil (Corbisier et al., 2006). The horizontal purple dashed line represents *δ*^15^N values of *T. testudinum* shoots from Almirante Bay and horizontal red solid line represents *δ*^15^N values of *T. testudinum* in Chiriqui Lagoon (Carruthers et al., 2005).

When trophic enrichment is considered (−3.4‰; Minagawa & Wada, 1984), *δ*^15^N values for Bocas del Toro bivalve tissue suggest a N source of <1.5‰, similar to or lower than the *δ*^15^N of natural N sources. Even considering lower *δ*^15^N values for septic-influenced sites, like those found in a study of lower Florida Keys macroalgae (3 to 5‰; Lapointe, Barile & Matzie, 2004), the evidence for significant sewage influence N at populated sites (Bocas Marina, Bocas Town, and the STRI Facility) is equivocal (Fig. 8). Although *δ*^15^N values at human-influenced sites are higher than those at uninfluenced sites, we have demonstrated that there are many potential sources that contribute to shell and tissue *δ*^15^N values, and these sources also vary across the region. In agreement with the wide variety of organic *δ*^13^C sources in the area, it is apparent that no single source dominates the *δ*^15^N signal in bivalve shells and tissues from the Bocas del Toro Archipelago, Panama.

There is a significant correlation between shell and tissue *δ*^15^N values in tropical pterioideans *P. imbricata* and *I. alatus* (Fig. 7), supporting the use of shell *δ*^15^N values as a proxy for tissue *δ*^15^N values. Small shell-tissue offsets (Δ^15^N_tissue-shell_ ≤ 1.2‰) may be partially attributed to the averaging of seasonal signal in shell organic matrix compared to the short-term *δ*^15^N recorded in tissues (Table 2). These findings agree with other studies that have used a different technique, acidification, to extract shell organic matrix from the CaCO_3_ (O’Donnell et al., 2003; Carmichael et al., 2008; Watanabe, Kodama & Fukuda, 2009; Versteegh, Gillikin & Dehairs, 2011). However, differences in Δ^15^N_tissue−shell_ values have been observed that are likely the result of irregular tissue turnover rates and seasonal variations in metabolism (Versteegh, Gillikin & Dehairs, 2011). Nevertheless, these studies and ours clearly show the utility of N isotopes in mollusk shells to examine nutrient sources and trophic structure in modern and past ecosystems.

**Table 2:** Average tissue-species offset (Δ*δ*^15^N_tissue-*shell*_ (%)) for various bivalve species. Table modified from Versteegh, Gillikin & Dehairs (2011).

Species	Δ*δ*^15^N_tissue-*shell*_ (‰)	Tissue type	Study
*Ruditapes philippinarum*	1.1 ± 0.4	Whole	Watanabe, Kodama & Fukuda, 2009
*Mercenaria mercenaria*	2.4 ± 0.3	Whole	Carmichael, Brenda & Valiela (2004)
*Mercenaria mercenaria*	0.2 ± 0.7	Mantle	O’Donnell et al. (2003)
*Arctica islandica*	2.7	Whole	LeBlanc (1989)
*Mytilus edulis*	−0.1 ± 0.2	Whole	LeBlanc (1989)
*Mytilus edulis*	−2.2 to −1.5	Mantle	Versteegh, Gillikin & Dehairs (2011)
*Pinctada imbricata*	0.0 ± 0.4	Muscle	This study
*Pinctada imbricata*	0.0 ± 0.5	Mantle	This study
*Isognomon alatus*	−0.3 ± 0.2	Muscle	This study
*Isognomon alatus*	−0.4 ± 0.2	Mantle	This study
*Brachidontes exustus*	0.2 ± 0.2	Muscle	This study
*Brachidontes exustus*	0.2 ± 0.3	Mantle	This study

The range of bivalve tissue *δ*^13^C values, about −20 to −16‰, encompasses average *δ*^13^C values for marine POM from the open Caribbean (−21 to −19‰; Eadie & Jeffrey, 1973; Fry et al., 1982) and Bocas del Toro Archipelago (−21.2 to −19.6‰; Seemann et al., 2014), and for grassland residue from Panama (−16.2‰; Schwendenmann & Pendall, 2006). Bivalve tissues in our study are enriched in ^13^C relative to Panamanian leaf litter (−30.1 ± 0.5‰; Schwendenmann & Pendall, 2006), banana leaf (-26.5‰; Hilbun, 2009), and *Rhizophora mangle* leaves and peat (−30.0 and −26.5‰ respectively; Hilbun, 2009) (Fig. 8). Conversely, tissues are depleted in ^13^C with respect to terrestrial grass (−12.1‰) and *Thalassia testudinum* (−8.5‰) from Almirante Bay (Hilbun, 2009). As might be predicted, the range of organic matter *δ*^13^C values from this study suggests a mixture of contributing sources, with no single source dominating. Escudo de Veraguas experiences the least terrestrial influence and thus the site’s high bivalve tissue *δ*^13^C values (−16.1 ± 0.4‰) relative to terrestrially-influenced sites such as Bocas Town (−18.9 ± 0.0‰) and Bocas Marina (−18.9 ± 0.8‰) suggest perhaps a greater influence from seagrass carbon. Indeed, seagrasses are dense on the large fringing reef surrounding the island. In contrast, the ^13^C depletion in bivalve tissue at Bocas Town and Bocas Marina sites likely reflects greater influence of mangrove carbon. Carbon isotope studies of seagrasses from Florida support this hypothesis, showing that mineralization of mangrove organic matter can significantly impact the *δ*^13^C of organic matter near mangrove forests (Lin, Banks & Sternberg, 1991; Anderson & Fourqurean, 2003). The lack of a distinctive terrestrial *δ*^13^C signature in the Rio Guarumo samples suggests that terrestrial organic carbon is not a dominant influence near the river mouth.

Oxygen isotope ratios (^18^O/^16^O) in shell carbonate provide a record of sea surface temperature (SST) and seawater *δ*^18^O, which covaries with salinity. Since annual variation in SST in the southwest Caribbean is low (∼2°C), the primary driver of variability in *δ*^18^O values is salinity (Tao et al., 2013). The *δ*^18^O values of bivalve shells in the Bocas del Toro Archipelago are consistent with the spatial variations in salinity. The open-ocean end-member Escudo de Veraguas has the highest average *δ*^18^O (−1.0‰) value and the freshwater end-member Rio Guarumo (Chiriquí Lagoon) shows the lowest *δ*^18^O (−1.7‰; average of *P. imbricata* and *B. exustus*). These results confirm our environmental interpretations and are consistent with the recently updated *δ*^18^O-salinity relationship for the southwest Caribbean which suggests that Escudo de Veraguas seawater (salinity 34.0‰) would be 0.25–0.5‰ higher in *δ*^18^O than Rio Guarumo seawater (salinity 31.6‰) (Tao et al., 2013). Isla Popa (−1.4‰) is enclosed within Chiriquí Lagoon showing characteristic freshwater influence similar to Rio Guarumo (D’Croz, Rosario & Gondóla, 2005). Bocas Town, Boca del Drago, STRI Lagoon (Matumbal), and Bocas Town Marina are semi-enclosed bodies of water and have shell *δ*^18^O values that lie closer to the marine end-member than the freshwater end-member. These transitional coastal sites are indistinguishable based on shell *δ*^18^O values.

## Conclusions

No single dominant source of organic matter contributes to *δ*^15^N and *δ*^13^C values in shallow-water mollusk tissues from Bocas del Toro. Mollusk *δ*^15^N and *δ*^13^C values are heavily influenced by local effects from mangrove and seagrasses, although the influence of C_3_ and C_4_ terrestrial N and C cannot be ruled out. Despite hydrographic differences indicated by *δ*^18^O values, there are only minor differences in *δ*^15^N and *δ*^13^C values between river-influenced end-member Rio Guarumo and open-ocean end-member Escudo de Veraguas, reinforcing the conclusion that C and N from the locally-abundant mangrove and seagrasses have relatively higher influence. High relative *δ*^15^N values are observed at populated sites, consistent with influences from sewage or septic waste, but the relatively low *δ*^15^N values overall (4.0–5.3‰) mean that the anthropogenic signature, if it exists, is dilute.

Shell *δ*^15^N values are a reliable proxy for organic matter *δ*^15^N values in bivalves in tropical coastal environments. There are strong correlations between the nitrogen isotopic compositions of tissue and shell organic matrix for *P. imbricata* data, *I. alatus* data, and combined *P. imbricata* and *I. alatus* data. Thus, for these species, N isotope studies of well-preserved historical and fossil shells should provide reliable records of nutrient sources and trophic structure in modern and past ecosystems.

##  Supplemental Information

10.7717/peerj.2278/supp-1 Appendix S1 Sample Collection Information

10.7717/peerj.2278/supp-2 Appendix S2 Species comparisons *Pinctada imbricata (P)*, *Brachidontes exustus (Br)*, *Isognomon alatus (I)*.

10.7717/peerj.2278/supp-3 Appendix S3 Summary of bivalve shell nitrogen, carbon and oxygen values. Duplicates are averaged. Dashes indicate no data is available for those samples.

10.7717/peerj.2278/supp-4 Appendix S4 Sample collection information for all specimens

## Additional Information and Declarations

### Field Study Permissions

The following information was supplied relating to field study approvals (i.e., approving body and any reference numbers): Collection permits were approved by the Autoridad de los Recursos Acuáticos de Panamá (permiso con fines científicos N°55).

### Data Availability

The following information was supplied regarding data availability: The data sets are supplied as supplementary data tables Appendices S1–S4.
