# Supplementary material for: Stable isotopes in bivalves as indicators of nutrient source in coastal waters in the Bocas del Toro Archipelago, Panama
Source: PeerJ. 2016 Aug 2;4:e2278. doi: 10.7717/peerj.2278 (PMC4975030; doi:10.7717/peerj.2278)
Supplement: Appendix S1 [file peerj-04-2278-s001.pdf]

Appendix I. Individual nitrogen and carbon isotopic analyses of bivalves. Sample IDs are coded by location (Escudo de Veraguas, EV, Boca del Drago, BD, STRI Facility, SF, Bocas Town Marina, BM, Isla Popa, LCN, Punta Sumwood, CA, Bocas Town, BT, Rio Guarumo, LCS) and by sample material (gill, g, stomach, s, mantle, ma, and muscle, mu). Duplicates are averaged.

ID	# ID	$\delta^{15}N$ vs. Air	$\delta^{13}C$ vs. VPDB
Pinctada imbricada			
EV13-P-4-s	97	2.87	-17.43
EV13-P-3-s	96	2.98	-16.35
EV13-P-4-mu	98	3.31	-15.86
EV13-P-3-mu	95	3.14	-15.73
EV13-P-4-g	100	3.32	-17.96
EV13-P-2-mu	102	2.80	-16.73
EV13-P-2-g	104	3.44	-16.72
EV13-P-2-s	101	2.52	-17.60
EV13-P-4-ma	99	3.15	-16.19
BD13-P-4-g	68	4.42	-17.65
BD13-P-4-ma	72	3.79	-17.51
BD13-P-5-s	77	3.43	-18.79
SF13-P-5-g	207	5.25	-17.86
SF13-P-5-ma	206	4.20	-17.79
SF13-P-5-mu	209	4.45	-17.44
SF13-P-5-s	208	3.92	-18.57
SF13-P-2-g	236	4.61	-17.83
SF13-P-2-ma	237	4.07	-18.22
SF13-P-2-mu	235	4.15	-17.42
SF13-P-2-s	234	4.03	-18.09
BM13-P-5-mu	263	5.45	-17.64
BM13-P-5-s	265	3.73	-19.63
BM13-P-5-ma	262	4.62	-18.97
BM13-I-4-s	251	2.79	-20.12
BM13-P-5-g	264	5.07	-19.00
BM13-P-3-s	279	4.91	-18.87
BM13-P-3-mu	282	5.75	-19.23
BM13-P-3--ma	281	5.49	-19.95
BM13-P-3--g	280	5.93	-20.00
LCN13-P-1-ma	109	3.49	-19.98
LCN13-P-2-s	122	3.02	-20.52
LCN13-P-2-g	119	3.96	-19.94
LCN13-P-2-ma	121	3.71	-20.05
LCN13-P-1-s	111	3.74	-18.32
CA13-P-5-s	156	2.62	-19.55
CA13-P-5-mu	159	3.10	-16.97
CA13-P-5-ma	158	2.86	-17.30
CA13-P-5-g	157	3.64	-17.40
CA13-P-4-s	177	3.22	-17.47
CA13-P-4-mu	179	3.89	-18.25
CA13-P-4-ma	176	3.60	-16.71
CA13-P-4-g	178	4.28	-18.58
BD13-P-4-s	20	4.12	-17.72
BD13-P-5-g	21	3.29	-18.09
BD13-P-4-mu	25	3.66	-17.25
BD13-P-3-s	60	3.49	-17.93
BD13-P-3-mu	65	3.83	-17.33
BD13-P-5-g	20	4.13	-17.80
BD13-P-4-s	21	3.11	-18.09
LCN13-P-1-mu	110	3.77	-17.42
BT13-P-2-mu	46	4.04	-18.89
BT-13-P-2-s	40	4.16	-19.89
BT13-P-2-mu	46	4.00	-18.91
	**Average**	**3.85**	**-18.14**
	**std dev**	**0.79**	**1.16**

ID	# ID	$\delta^{15}N$ vs. Air	$\delta^{13}C$ vs. VPDB
Isognomon alatus			
SF13-I-4-s	219	3.78	-18.97
SF13-I-4-ma	220	4.28	-18.67
SF13-I-4-mu	218	4.26	-17.68
SF13-I-5-s	224	3.54	-18.96
SF13-I-5-g	225	4.48	-18.55
SF13-I-5-ma	223	4.12	-18.43
SF13-I-5-ma	223	4.17	-18.51
SF13-I-4-g	221	4.60	-18.56
SF13-I-5-mu	222	3.99	-17.93
BM13-I-4-ma	253	4.03	-19.45
BM13-I-4-g	252	4.43	-19.25
BM13-I-2-mu	258	4.83	-18.09
BM13-I-4-mu	250	4.08	-18.62
BM13-I-2-g	259	4.70	-19.03
BM13-I-2-ma	261	4.39	-19.10
BM13-I-2-s	260	3.48	-20.15
LCN13-I-4-ma	134	3.85	-19.95
LCN13-I-4-s	133	3.20	-20.70
LCN13-I-1-ma	138	4.06	-19.77
LCN13-I-1-s	139	3.40	-20.80
LCN13-I-4-mu	131	4.01	-19.24
LCN13-I-4-g	132	3.78	-21.84
LCN13-I-1-g	140	4.30	-17.68
LCN13-I-1-mu	141	3.98	-17.24
CA13-I-4-g	169	3.61	-17.45
CA13-I-4-s	168	2.85	-19.73
CA13-I-3-mu	173	3.37	-16.73
CA13-I-4-mu	170	3.24	-17.02
CA13-I-3-ma	174	3.33	-17.41
CA13-I-3-g	175	3.60	-17.44
CA13-I-4-ma	171	3.41	-17.60
CA13-I-3-s	172	2.83	-19.60
BD13-I-3-s	4	3.02	-19.23
BD13-I-1-g	7	4.09	-18.60
BD13-P-3-g	8	4.49	-17.75
BD13-I-2-s	13	3.40	-19.94
BD13-I-2-ma	15	3.77	-19.32
BD13-I-2-g	16	3.81	-20.20
BD13-I-1-s	17	3.41	-19.30
BD13-I-2-mu	18	3.41	-18.62
BD13-I-1-mu	19	3.63	-17.97
BD13-I-1-ma	27	4.54	-19.10
BD13-I-1-g	7	4.05	-18.76
BD13-I-2-s	13	3.11	-20.11
BD13-I-2-ma	15	3.67	-19.29
BD13-I-2-g	16	3.67	-20.07
BD13-I-1-s	17	3.17	-19.19
BD13-I-2-mu	18	3.58	-18.69
BD13-I-1-mu	19	3.65	-17.94
BD13-I-2-ma	15	3.66	-17.29
BD13-I-1-ma	27	4.56	-19.40
BD13-I-2-s	39	2.85	-21.98
	**Average**	**3.80**	**-18.90**
	**std dev**	**0.50**	**1.14**

ID	ID #	$\delta^{15}N$ vs. Air	$\delta^{13}C$ vs. VPDB
Brachidontes exustus			
LCS13-Br-1-ma	200	3.56	-17.78
LCS13-Br-1-mu	201	3.68	-16.91
LCS13-Br-5-ma	199	4.30	-20.02
LCN13-Br-3-ma	115	3.98	-20.17
LCN13-Br-3-mu	116	3.93	-19.17
LCN-13-Br-1-mu	113	3.68	-19.41
BD13-Br-2-mu	23	3.49	-18.53
BD13-Br-2-ma	24	3.39	-20.06
BD13-Br-2-mu	23	3.54	-18.26
BD13-Br-2-ma	24	3.26	-20.02
BD13-Br-3-mu	35	3.30	-18.59
BD13-Br-3-ma	59	3.11	-20.90
LCS13-Br-5-mu	198	3.99	-16.89
	**Average**	**3.63**	**-18.98**
	**std dev**	**0.33**	**1.23**
