# Supplementary material for: Stable isotopes in bivalves as indicators of nutrient source in coastal waters in the Bocas del Toro Archipelago, Panama
Source: PeerJ. 2016 Aug 2;4:e2278. doi: 10.7717/peerj.2278 (PMC4975030; doi:10.7717/peerj.2278)
Supplement: Appendix S2 — Pinctada imbricata (P), Brachidontes exustus (Br), Isognomon alatus (I). [file peerj-04-2278-s002.pdf]

Appendix II.

**Location**	**Comparison**	**P value ($\delta^{15}N$)**	**P value ($\delta^{13}C$)**
Isla Popa	P vs. I	**0.03**	0.90
	P vs. Br	0.18	0.67
	Br vs. I	0.35	0.50
Boca del Drago	P vs. I	0.84	**0.01**
	P vs. Br	**0.01**	**0.03**
	Br vs. I	0.07	0.23
STRI Facility	P vs. I	0.45	0.41
Bocas Marina	P vs. I	**0.02**	0.48
Punta Sumwood	P vs. I	0.84	0.75
