# Supplementary material for: Stable isotopes in bivalves as indicators of nutrient source in coastal waters in the Bocas del Toro Archipelago, Panama
Source: PeerJ. 2016 Aug 2;4:e2278. doi: 10.7717/peerj.2278 (PMC4975030; doi:10.7717/peerj.2278)
Supplement: Appendix S3 — Summary of bivalve shell nitrogen, carbon and oxygen values. Duplicates are averaged. Dashes indicate no data is available for those samples. [file peerj-04-2278-s003.pdf]

Appendix III. Summary of bivalve shell nitrogen, carbon, and oxygen values. Species names are coded as: *Pinctada imbricata*, P, *Isognomon alatus*, I, *Brachidontes exustus*, Br. Duplicates are averaged. Dashes indicate no data is available for those samples.

**Location**	**Shell ID**	**Species**	**$\delta^{15}N$ (‰ vs. air)**	**$\delta^{13}C$ (‰ vs. VPDB)**	**$\delta^{18}O$ (‰ vs. VPDB)**	**%N**
Bocas Town	BT1	P	3.62	-0.96	-1.21	0.008
Bocas Town	BT2	P	4.16	-0.97	-1.25	0.009
Bocas Town	BT3	P	4.75	-0.87	-1.08	0.011
Bocas Town	BT4	P	4.92	-0.88	-1.06	0.009
Bocas Town	BT5	P	5.22	-0.69	-0.95	0.008
Bocas Town	BT6	I	5.15	0.02	-1.20	0.005
Bocas Town	BT7	I	4.71	-0.02	-1.16	0.007
Bocas Town	BT8	I	4.93	-	-	0.006
Bocas Town	BT9	I	2.87	1.81	-1.35	0.010
Bocas Town	BT10	I	4.84	-0.36	-1.09	0.006
Escudo de Veraguas	EV1	P	3.04	-1.42	-1.09	0.006
Escudo de Veraguas	EV2	P	3.16	-0.56	-0.91	0.005
Escudo de Veraguas	EV3	P	3.22	0.14	-1.10	0.005
Escudo de Veraguas	EV4	P	3.03	0.08	-1.16	0.006
Escudo de Veraguas	EV5	P	3.60	-1.37	-0.94	0.006
Rio Guarumo	LCS1	P	4.80	-	-	0.008
Rio Guarumo	LCS2	P	4.87	-0.32	-2.00	0.045
Rio Guarumo	LCS3	Br	3.92	-0.03	-1.67	0.046
Rio Guarumo	LCS4	Br	4.27	-0.01	-1.59	0.057
Rio Guarumo	LCS5	Br	4.34	-0.02	-1.50	0.054
Rio Guarumo	LCS6	Br	3.98	0.25	-1.40	0.045
Rio Guarumo	LCS7	Br	4.69	0.18	-1.05	0.091
STRI Facility	SF1	I	3.70	-0.14	-1.37	0.007
STRI Facility	SF2	I	3.72	-0.02	-1.10	0.007
STRI Facility	SF3	I	3.89	-	-	0.007
STRI Facility	SF4	I	3.79	-0.20	-1.00	0.008
STRI Facility	SF5	I	3.59	0.03	-1.09	0.010
STRI Facility	SF6	P	3.72	-1.16	-1.58	0.008
STRI Facility	SF7	P	3.39	-0.96	-1.21	0.009
STRI Facility	SF8	P	3.80	-0.53	-0.95	0.007
STRI Facility	SF9	P	3.67	-1.15	-1.40	0.007
STRI Facility	SF10	P	3.68	-1.04	-1.16	0.006
Isla Popa	LCN1	I	3.25	0.92	-1.53	0.009
Isla Popa	LCN2	I	3.44	0.29	-1.43	0.008
Isla Popa	LCN3	I	2.68	0.62	-1.54	0.008
Isla Popa	LCN4	I	2.74	0.52	-1.50	0.008
Isla Popa	LCN5	I	2.91	0.48	-1.37	0.008
Isla Popa	LCN6	P	3.05	-1.09	-1.40	0.009
Isla Popa	LCN7	P	3.40	-1.19	-1.42	0.007
Isla Popa	LCN8	P	3.24	-1.05	-1.41	0.007
Isla Popa	LCN9	P	3.41	-1.11	-1.35	0.008
Isla Popa	LCN10	Br	3.84	-0.33	-1.46	0.047
Isla Popa	LCN11	Br	3.87	-0.28	-1.23	0.038
Isla Popa	LCN12	Br	3.58	-0.26	-1.45	0.023
Bocas Town Marina	BM1	P	5.37	-1.67	-1.22	0.009
Bocas Town Marina	BM2	P	5.65	-1.15	-1.14	0.010
Bocas Town Marina	BM3	P	5.11	-1.25	-1.07	0.012
Bocas Town Marina	BM4	P	4.52	-1.26	-1.30	0.014
Bocas Town Marina	BM5	P	5.39	-1.50	-1.26	0.011
Bocas Town Marina	BM6	I	4.75	-0.64	-1.51	0.009
Bocas Town Marina	BM7	I	4.25	-1.01	-1.38	0.010
Bocas Town Marina	BM8	I	4.22	-0.87	-1.32	0.009
Bocas Town Marina	BM9	I	3.45	-1.04	-1.58	0.011
Bocas Town Marina	BM10	I	4.14	-0.65	-1.49	0.009
Punta Sumwood	CA1	I	2.93	0.36	-1.34	0.007
Punta Sumwood	CA2	I	3.25	0.43	-1.18	0.007
Punta Sumwood	CA3	I	2.10	0.57	-1.24	0.007
Punta Sumwood	CA4	I	1.45	0.78	-1.22	0.009
Punta Sumwood	CA5	I	2.78	0.74	-1.18	0.007
Punta Sumwood	CA6	P	3.07	-0.63	-1.34	0.008
Punta Sumwood	CA7	P	3.08	-0.61	-1.42	0.008
Punta Sumwood	CA 8	P	3.14	-0.49	-1.19	0.007
Punta Sumwood	CA 9	P	2.93	-0.64	-1.13	0.008
Punta Sumwood	CA 10	P	3.19	-0.37	-1.19	0.006
Boca del Drago	BD 1	I	3.98	-0.69	-1.30	0.006
Boca del Drago	BD 2	I	4.01	-0.46	-1.27	0.005
Boca del Drago	BD 3	I	3.79	-0.63	-1.22	0.007
Boca del Drago	BD 4	I	3.06	0.35	-1.37	0.006
Boca del Drago	BD 5	I	3.67	-0.64	-1.31	0.004
Boca del Drago	BD 6	P	3.17	-0.51	-0.98	0.006
Boca del Drago	BD 7	P	3.50	-0.35	-1.00	0.005
Boca del Drago	BD 8	P	3.59	-0.52	-1.21	0.004
Boca del Drago	BD 9	P	3.45	-	-	0.008
Boca del Drago	BD 10	P	3.72	-0.54	-1.15	0.006
Boca del Drago	BD 11	Br	3.63	-0.58	-1.09	0.067
Boca del Drago	BD 12	Br	3.27	-0.64	-1.30	0.077
Boca del Drago	BD 13	Br	3.45	-0.39	-1.13	0.085
