# Supplementary material for: Stable isotopes in bivalves as indicators of nutrient source in coastal waters in the Bocas del Toro Archipelago, Panama
Source: PeerJ. 2016 Aug 2;4:e2278. doi: 10.7717/peerj.2278 (PMC4975030; doi:10.7717/peerj.2278)
Supplement: Appendix S4 [file peerj-04-2278-s004.pdf]

Appendix IV. Sample collection information.

Species	Shell ID #	Depth	Max diam./width (mm)	Height (mm)	Comments
**Boca del Drago, 9°23.652' N, 82°18.668' W (collected 7/19/2013)**					
Isognoman alatus	BD13-I-1	<1 m	42	48	mangroves
Isognoman alatus	BD13-I-2	<1 m	34	42	mangroves
Isognoman alatus	BD13-I-3	<1 m	40	42	mangroves
Isognoman alatus	BD13-I-4	<1 m	34	29	mangroves
Isognoman alatus	BD13-I-5	<1 m	46	46	mangroves
Pinctada umbricata	BD13-P-1	<1 m	42	44	mangroves
Pinctada umbricata	BD13-P-2	<1 m	42	40	mangroves
Pinctada umbricata	BD13-P-3	<1 m	46	48	mangroves
Pinctada umbricata	BD13-P-4	<1 m	50	53	mangroves
Pinctada umbricata	BD13-P-5	<1 m	52	57	mangroves
Brachidontes exustus	BD-Br-1	<1 m	16	7	mangroves
Brachidontes exustus	BD-Br-2	<1 m	18	8	mangroves
Brachidontes exustus	BD-Br-3	<1 m	17	7	mangroves
**Bocas Town, 9°20.463' N, 82°14.480' W (collected 7/19/2013)**					
Pinctada umbricata	BT13-P-1	<1 m	32	31	off dock, deeper water here, septic tanks (no direct sewage input?)
Pinctada umbricata	BT13-P-2	<1 m	30	28	off dock, deeper water here, septic tanks (no direct sewage input?)
Pinctada umbricata	BT13-P-3	<1 m	40	36	off dock, deeper water here, septic tanks (no direct sewage input?)
Pinctada umbricata	BT13-P-4	<1 m	43	44	off dock, deeper water here, septic tanks (no direct sewage input?)
Pinctada umbricata	BT13-P-5	<1 m	45	46	off dock, deeper water here, septic tanks (no direct sewage input?)
Isognoman alatus	BT13-I-1	<1 m	35	33	off dock, deeper water here, septic tanks (no direct sewage input?)
Isognoman alatus	BT13-I-2	<1 m	30	29	off dock, deeper water here, septic tanks (no direct sewage input?)
Isognoman alatus	BT13-I-3	<1 m	32	36	off dock, deeper water here, septic tanks (no direct sewage input?)
Isognoman alatus	BT13-I-4	<1 m	29	31	off dock, deeper water here, septic tanks (no direct sewage input?)
Isognoman alatus	BT13-I-5	<1 m	31	27	off dock, deeper water here, septic tanks (no direct sewage input?)
**Escudo de Veraguas, 9°06.399' N, 81°33.318' W (collected 7/20/2013)**					
Pinctada umbricata	EV13-P-1	<1 m	40	39	mangroves
Pinctada umbricata	EV13-P-2	<1 m	34	37	mangroves
Pinctada umbricata	EV13-P-3	<1 m	20	27	mangroves
Pinctada umbricata	EV13-P-4	<1 m	55	47	mangroves
Pinctada umbricata	EV13-P-5	<1 m	56	64	mangroves
**Bocas Marina, 9°20.246' N, 82°14.707' W (collected 7/21/2013)**					
Pinctada umbricata	BM13-P-1	<1 m	37	36	off dock & mangroves, directly under outhouse
Pinctada umbricata	BM13-P-2	<1 m	39	39	off dock & mangroves, directly under outhouse
Pinctada umbricata	BM13-P-3	<1 m	22	25	off dock & mangroves, directly under outhouse
Pinctada umbricata	BM13-P-4	<1 m	36	36	off dock & mangroves, directly under outhouse
Pinctada umbricata	BM13-P-5	<1 m	26	21	off dock & mangroves, directly under outhouse
Isognoman alatus	BM13-I-1	<1 m	32	28	off dock & mangroves, directly under outhouse
Isognoman alatus	BM13-I-2	<1 m	40	47	off dock & mangroves, directly under outhouse
Isognoman alatus	BM13-I-3	<1 m	35	36	off dock & mangroves, directly under outhouse
Isognoman alatus	BM13-I-4	<1 m	35	35	off dock & mangroves, directly under outhouse
Isognoman alatus	BM13-I-5	<1 m	30	30	off dock & mangroves, directly under outhouse
**STRI Facility, 9°21'3.80"N, 82°15'26.01"W (collected 7/18/2013)**					
Pinctada umbricata	SF13-P-1	<1 m	48	43	off dock
Pinctada umbricata	SF13-P-2	<1 m	40	41	off dock
Pinctada umbricata	SF13-P-3	<1 m	32	40	off dock
Pinctada umbricata	SF13-P-4	<1 m	34	33	off dock
Pinctada umbricata	SF13-P-5	<1 m	36	40	off dock
Isognoman alatus	SF13-I-1	<1 m	44	49	off dock
Isognoman alatus	SF13-I-2	<1 m	22	25	off dock
Isognoman alatus	SF13-I-3	<1 m	49	47	off dock
Isognoman alatus	SF13-I-4	<1 m	32	36	off dock
Isognoman alatus	SF13-I-5	<1 m	45	41	off dock
**Cayo Adriana, 9°07.729' N, 82°09.197' W (collected 7/21/2013)**					
Isognoman alatus	CA13-I-1	<1 m	30	27	mangroves
Isognoman alatus	CA13-I-2	<1 m	30	28	mangroves
Isognoman alatus	CA13-I-3	<1 m	36	37	mangroves
Isognoman alatus	CA13-I-4	<1 m	37	30	mangroves
Isognoman alatus	CA13-I-5	<1 m	42	41	mangroves
Pinctada umbricata	CA13-P-1	<1 m	26	26	mangroves
Pinctada umbricata	CA13-P-2	<1 m	25	24	mangroves
Pinctada umbricata	CA13-P-3	<1 m	23	24	mangroves
Pinctada umbricata	CA13-P-4	<1 m	25	25	mangroves
Pinctada umbricata	CA13-P-5	<1 m	27	28	mangroves
**Rio Guarumo (Chiriqui Lagoon South), 8°59.965' N, 82°10.7567' W (collected 7/21/2013)**					
Pinctada umbricata	LCS13-P-1	<1 m	17	15	no gills; mangroves
Pinctada umbricata	LCS13-P-2	<1 m	23	19	gills and mantle together; mangroves
Brachidontes exustus	LCS13-Br-1	<1 m	9	5	mangroves
Brachidontes exustus	LCS13-Br-2	<1 m	12	6	mangroves
Brachidontes exustus	LCS13-Br-3	<1 m	13	7	mangroves
Brachidontes exustus	LCS13-Br-4	<1 m	11	6	mangroves
Brachidontes exustus	LCS13-Br-5	<1 m	7	6	mangroves
**Isla Popa (Chiriqui Lagoon North), 9°08.255 N,82°09.006 W (collected 7/21/2013)**					
Isognoman alatus	LCN13-I-1	<1 m	40	42	mangroves
Isognoman alatus	LCN13-I-2	<1 m	50	48	mangroves
Isognoman alatus	LCN13-I-3	<1 m	45	42	mangroves
Isognoman alatus	LCN13-I-4	<1 m	42	45	mangroves
Isognoman alatus	LCN13-I-5	<1 m	49	42	mangroves
Pinctada umbricata	LCN13-P-1	<1 m	28	29	mangroves
Pinctada umbricata	LCN13-P-2	<1 m	25	28	mangroves
Pinctada umbricata	LCN13-P-3	<1 m	14	13	mangroves
Pinctada umbricata	LCN13-P-4	<1 m	34	31	mangroves
Brachidontes exustus	LCN13-Br-1	<1 m	12	6	mangroves
Brachidontes exustus	LCN13-Br-2	<1 m	13	6	mangroves
Brachidontes exustus	LCN13-Br-3	<1 m	13	7	mangroves
